# Tracheal nodularity and paratracheal soft tissue nodule: post-radioactive iodine treatment changes with peculiar visual and pathologic findings in a case of metastatic follicular variant papillary thyroid carcinoma: a case report

**DOI:** 10.1186/s13256-025-05116-2

**Published:** 2025-03-03

**Authors:** Benjamin L. Sievers, Wangpan Shi, Jingjing Hu, Russell Miller, Niral M. Patel, Keriann Van Nostrand, George Z. Cheng, Charles H. Choe, Jorge Alberto Muñoz Pineda

**Affiliations:** 1 Infectious Diseases, J. Craig Venter Institute, La Jolla, CA 92037 USA; 2 Department of Medicine, University of Cambridge, Cambridge, UK; 3 Department of Pathology, University of California San Diego, La Jolla, CA 92037 USA; 4 Interventional Pulmonology, Department of Medicine, University of California San Diego, La Jolla, CA 92037 USA; 5 Department of Endocrinology, University of California San Diego, La Jolla, CA 92037 USA

**Keywords:** Thyroid malignancy, Papillary thyroid carcinoma, Thyroid cancer, Case report, Metastasis, Radioactive iodine, Nodules of the trachea, Tracheal nodularity, Bronchoscopy, Endobronchial ultrasound-guided sampling

## Abstract

**Background:**

Follicular variant papillary thyroid carcinoma is a distinct subtype of papillary thyroid carcinoma that can occasionally present with aggressive features, including distant metastases and extrathyroidal extension. While radioactive iodine ablation is a well-established treatment for residual disease, its post-treatment effects on tracheal and paratracheal structures remain poorly characterized.

**Case presentation:**

A 22-year-old male individual of Taiwanese descent presented with an enlarged neck mass and was diagnosed with follicular variant papillary thyroid carcinoma. He underwent thyroidectomy, modified radical neck dissection, and postoperative radioactive iodine-131 ablation (100 mCi). A total of 1 year later, a chest computed tomography revealed a paratracheal soft tissue nodule and tracheal nodularity. Bronchoscopy with endobronchial ultrasound-guided sampling identified multiple 2–3 mm submucosal tracheal nodules containing white exudate. Cytopathologic analysis of both the paratracheal soft tissue nodule and the tracheal wall nodules revealed mucinous material without evidence of malignancy or inflammation. Microbiologic studies were negative for infection.

**Conclusion:**

These atypical bronchoscopic and pathologic findings likely represent post-radioactive iodine treatment changes. The patient remained without evidence of disease for 22 months, ongoing on thyroid suppression levels of thyroxine hormone replacement. The case represents successful radioactive iodine treatment of papillary thyroid carcinoma residual disease after surgical resection, with the first described pathologic findings to correlate with these post-treatment changes.

## Background

Papillary thyroid carcinoma (PTC) is one of the most common cancers in young adults and the most common malignant thyroid neoplasm, with an average age of presentation of 50 years [1, 2]. In addition, thyroid cancer is among the most common malignancies in adolescents and adults between the ages of 16–33 years [3]. At diagnosis, PTC is commonly confined to the thyroid gland and regional lymph nodes; however, distant metastasis occur in 1–4% of cases [4]. The primary mode of PTC spread is lymphatic, as blood-borne metastasis is uncommon [5]. While PTC metastasis is well-defined, with distant spread mostly occurring in the bone, lung, skin, or brain, tracheal metastases are extremely rare but have been noted to occur on occasion [6].

Tracheal invasion from PTC is exceedingly rare as tracheal metastasis occurs through infiltration [7, 8]. Previous characterizations of endoluminal tracheal metastasis have varied, with some patients presenting with asymptomatic disease to dyspnea and hemoptysis [9]. Surgical resection or endoscopic removal paired with radioactive iodine is the main course of intervention for this rare pathology [8].

In this study, to the best of our knowledge, we describe the first pathologic findings of post-radioactive iodine treatment of PTC disease. This case is worth reporting as the endotracheal nodule findings were atypical and post-treatment pathology is unreported in literature.

## Case presentation

A 22-year-old male individual of Taiwanese descent noticed a sizable 11 mm short axis rubbery mass behind the sternocleidomastoid at the base of the clavicle and visited the University of California San Diego hospital for examination in 2022. The patient had one family member who presented with papillary thyroid carcinoma in 2016. Examination via ultrasonography revealed findings suggestive of pathologic lymphadenopathy in the right neck, along with a 5 × 6 mm mass detected in the right thyroid lobe on initial imaging studies. Following these observations, the patient was referred for further evaluation and management. A subsequent needle biopsy of the right neck level 4 lymph node of size 2.0 × 1.4 cm revealed histopathological features consistent with infiltrating follicular variant of papillary thyroid carcinoma, with focal tall cell features. The tall cell features, however, did not exceed 30% of the tumor; therefore, the tumor was not considered tall cell variant. Overall, the tumor demonstrated histopathological features consistent with metastatic follicular variant papillary thyroid carcinoma.

The patient underwent a complete thyroidectomy and right modified radical neck dissection. Pathology demonstrated papillary thyroid carcinoma of the resected thyroid with negative margins, though the margin was less than 1 mm in some areas. There was lymphovascular invasion and locoregional lymph node involvement on the right. Postoperatively, nuclear imaging demonstrated foci of activity in the lungs and in the anterior neck thyroid bed that were treated with radioactive iodine-131 at 100 mCi to clear remnant disease.

**Fig. 1:**
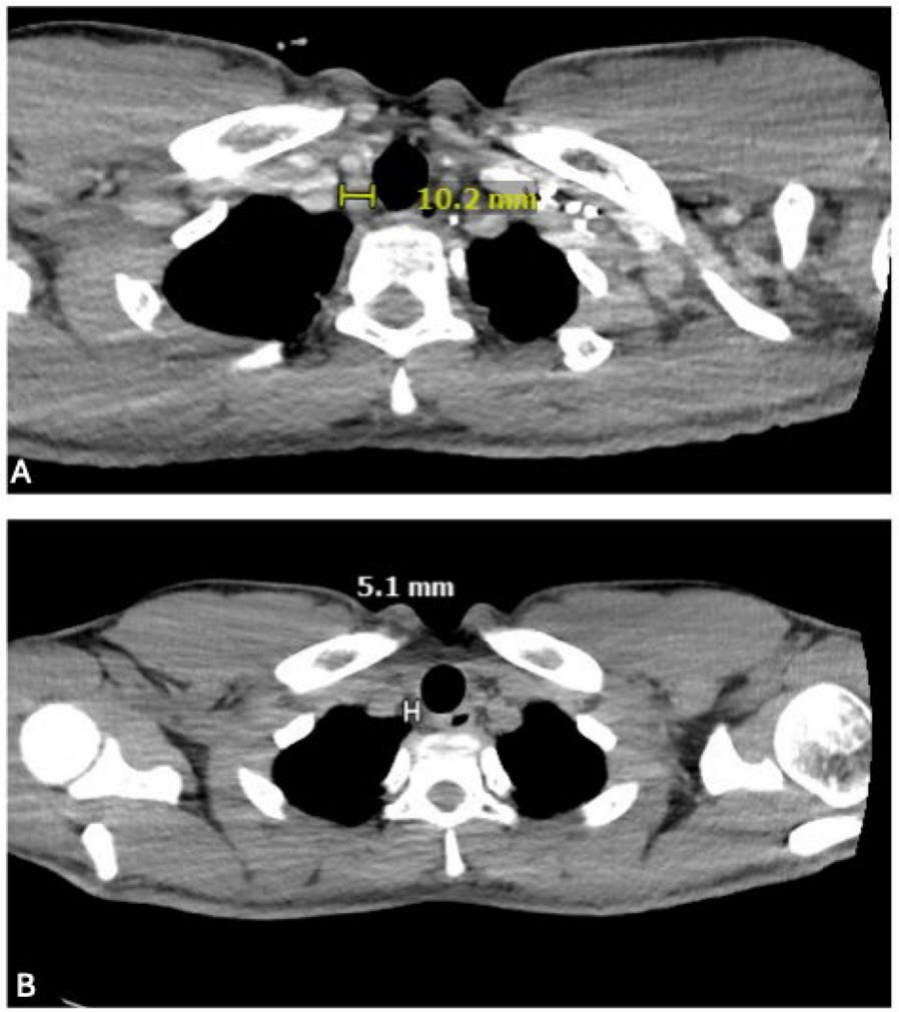
**A** Computed tomography with contrast chest at time of diagnosis showing 10 mm right paratracheal mass. **B** A total of 1 year post-treatment, surveillance computed tomography chest revealed a 5 mm right paratracheal soft tissue nodule at site of previous mass

**Fig. 2:**
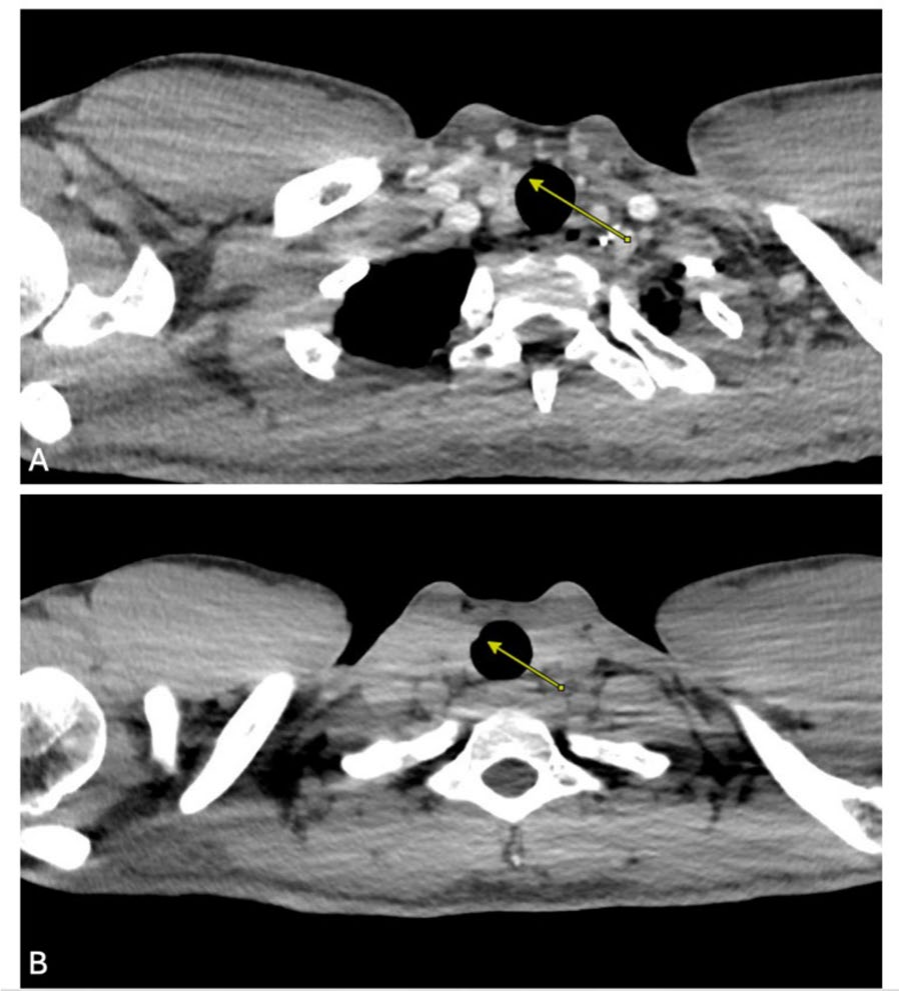
**A** Preoperative computed tomography with contrast chest showing tracheal nodularity. **B** A total of 1 year post-treatment, demonstrating persistent nodularity. Yellow arrow denotes area with persistant nodularity

Post-treatment surveillance imaging with computed tomography (CT) of the chest 1 year after treatment revealed a soft tissue nodule in the paratracheal thyroid bed (Fig. 1) and anterior tracheal wall nodularity, (Fig. 2) for which a bronchoscopy was pursued.

**Fig. 3:**
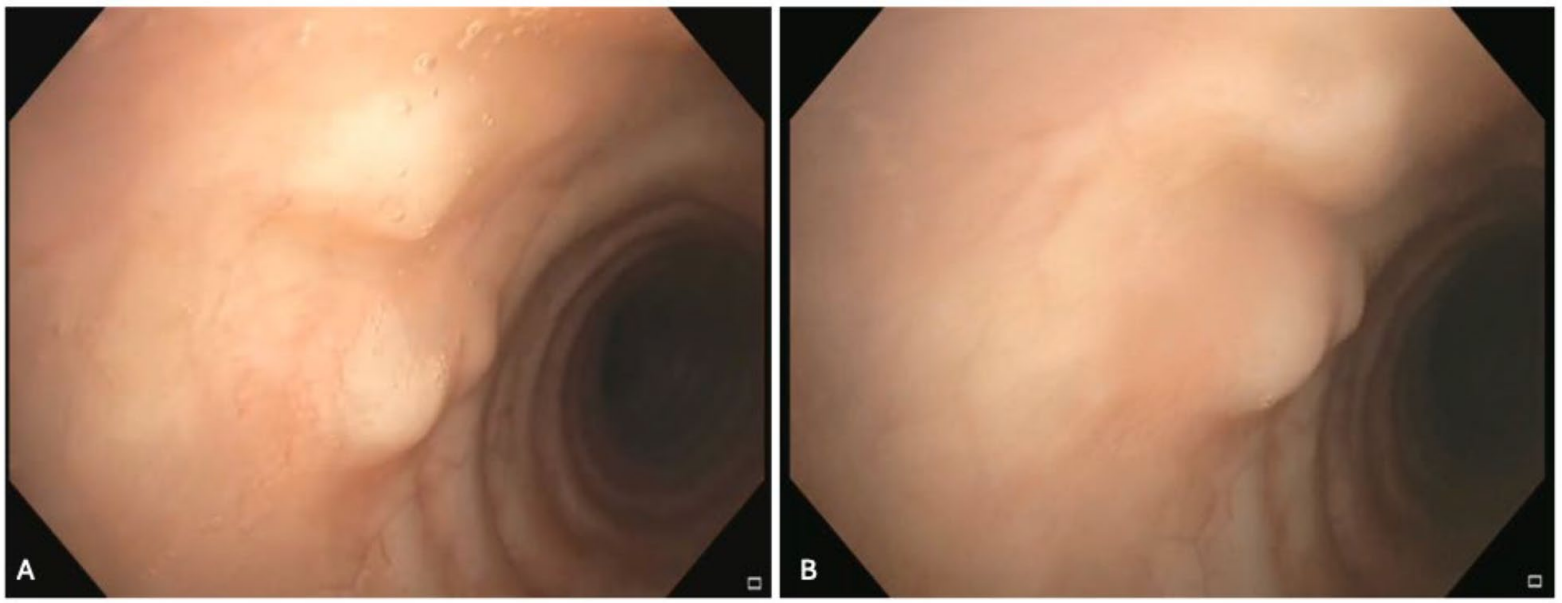
Bronchoscopic examination revealed a cluster of 2–3 mm nodules along anterior tracheal wall. **A** Nodules were covered by intact mucosa. **B** Different perspective of nodules illustrating intact and normal vascularity under narrow-band imaging

Bronchoscopic examination was notable for a cluster of small (2–3 mm) nodules along the right anterior tracheal wall (Fig. 3). These nodules were covered by intact mucosa and displayed normal vascularity under narrow-band imaging (NBI). Further evaluation using radial endobronchial ultrasound (EBUS) revealed a heterogeneous echotexture within the nodules.

**Fig. 4:**
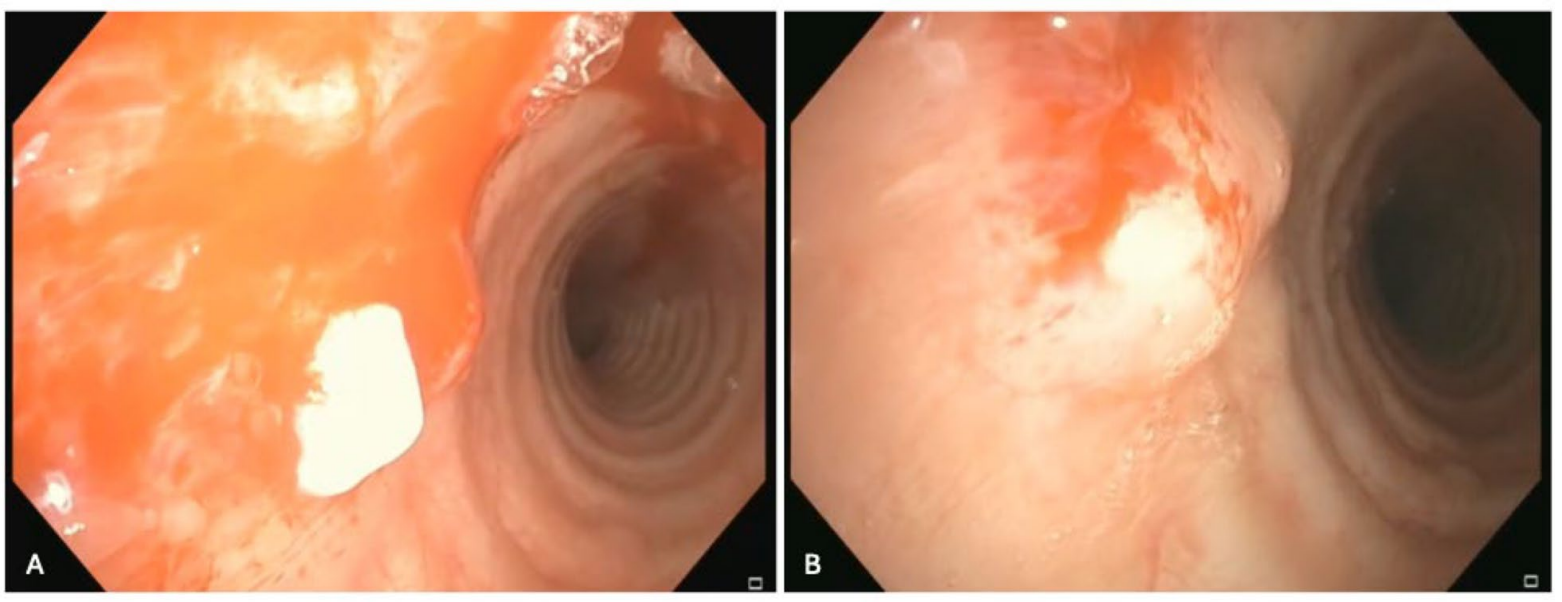
**A** Endobronchial biopsy of the tracheal nodules revealed a white exudate with soft density emanating from the submucosa. **B** Another angle of the nodules during unroofing

Endobronchial biopsy of the tracheal nodules was executed using a combination of alligator forceps and 1.1 mm cryoprobe. Remarkably, upon unroofing the tracheal nodules, a white exudate with soft density was observed emanating from the submucosa (Fig. 4). Tissue samples were forwarded for comprehensive microbiological and pathological analyses.

**Fig. 5:**
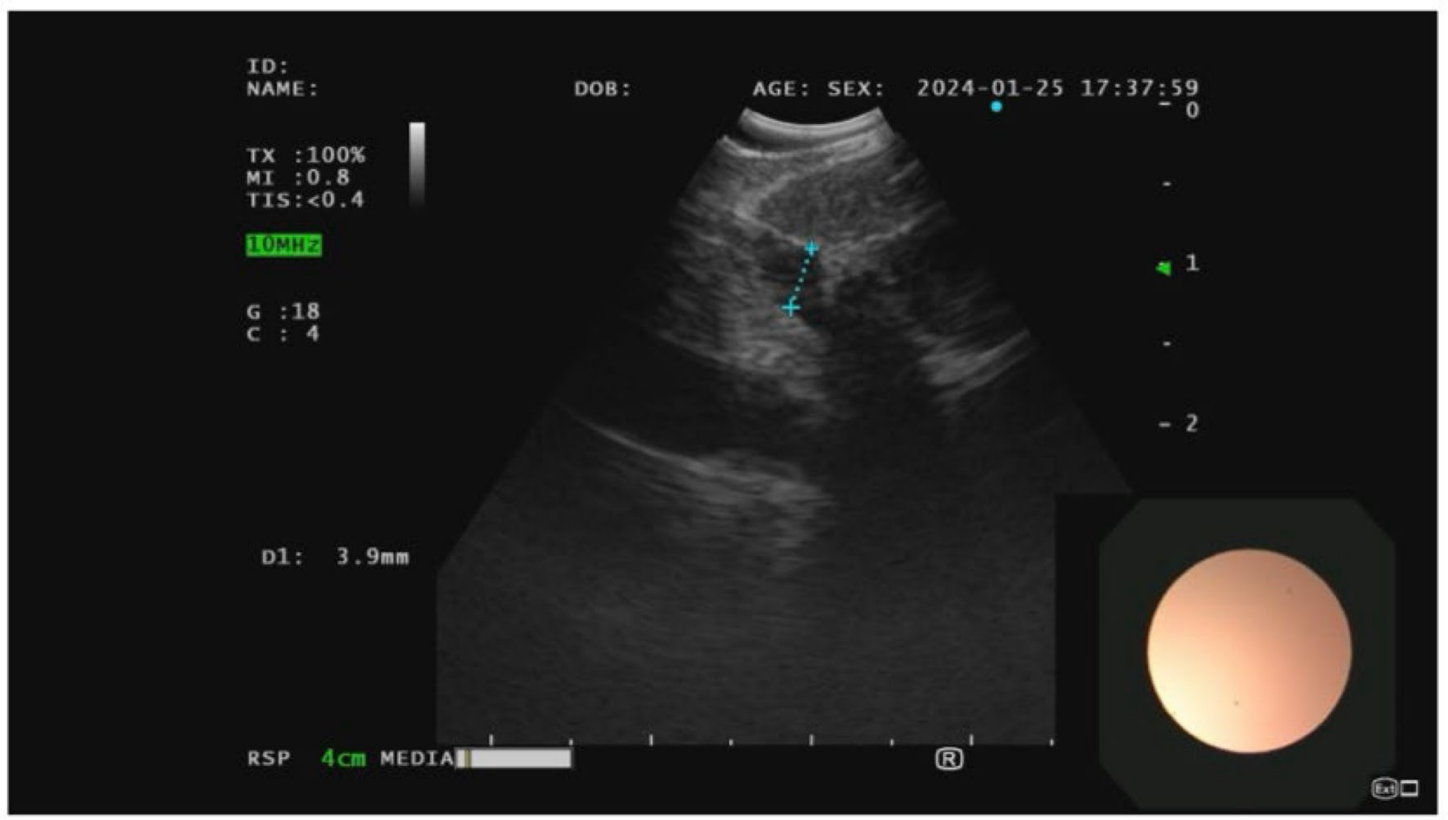
Linear endobronchial ultrasound of the paratracheal soft tissue nodule

**Fig. 6:**
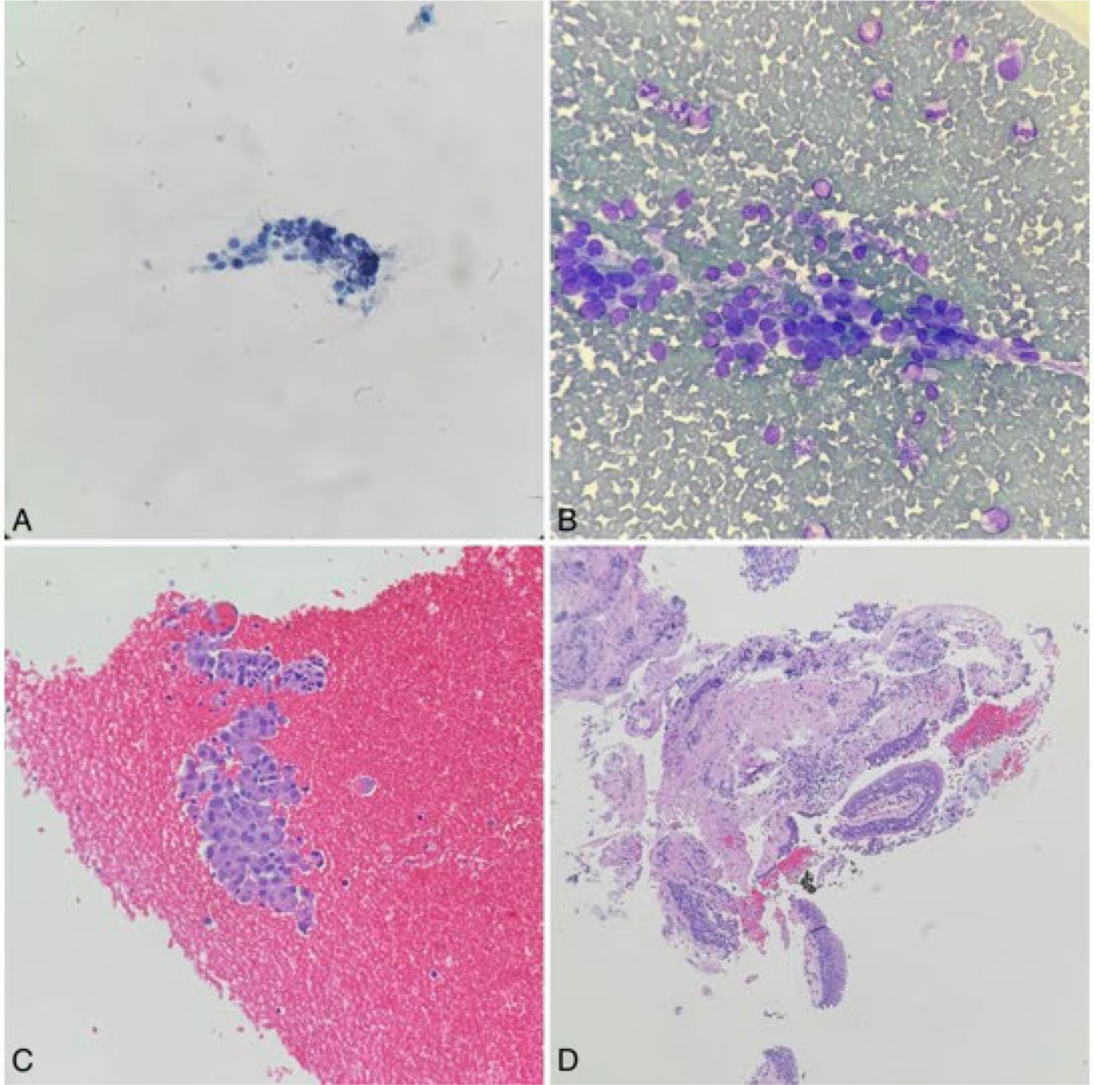
Pathology stains of endobronchial biopsy. **A**, **B** Pap smear and Diff-Quick sections showing clusters of follicular cells with crowding and overlapping nuclei with grooves and pallor, **C** cell block showing clusters of enlarged cells with nuclei pallor, **D** endobronchial biopsy showing benign respiratory epithelium with inflammation and fibrosis

**Fig. 7:**
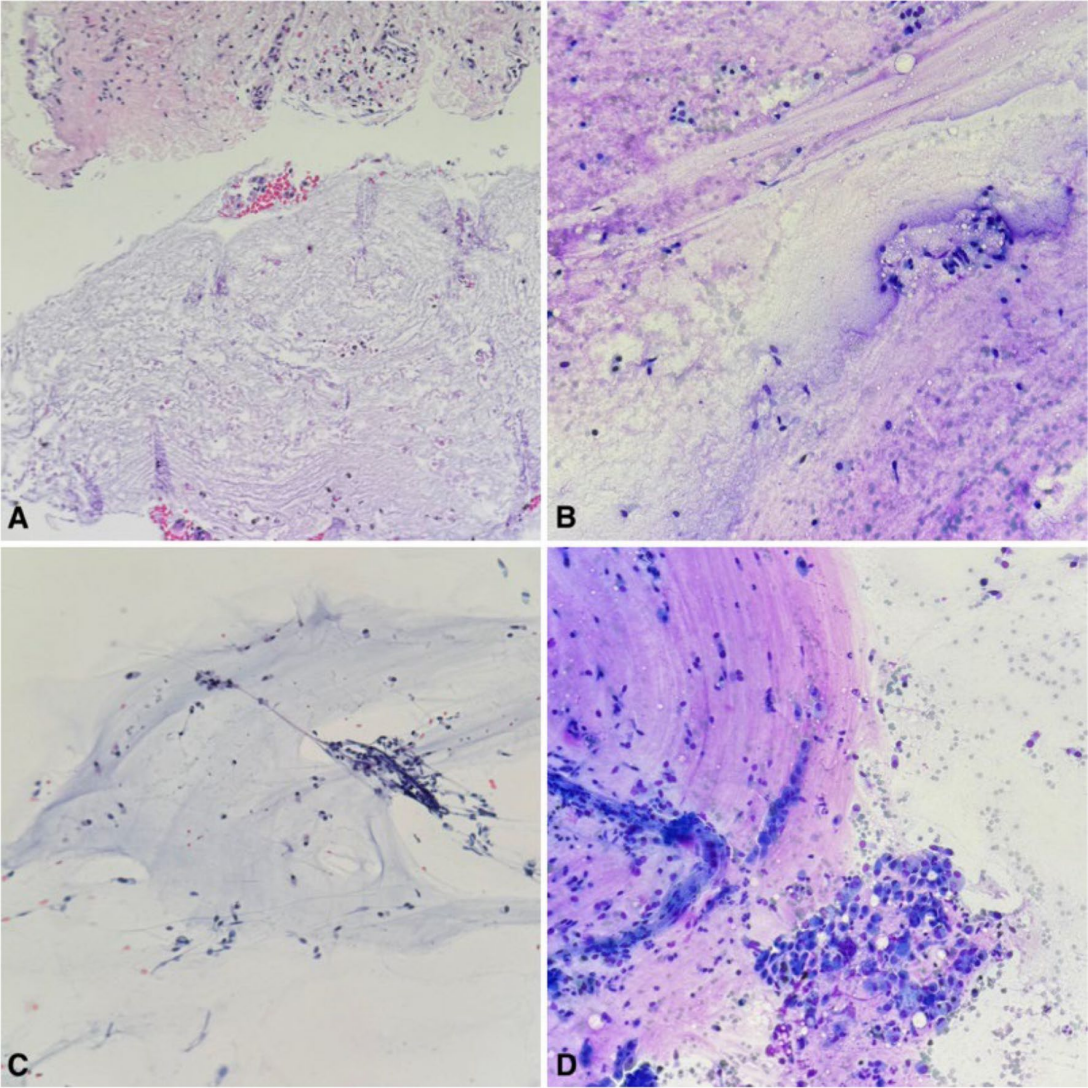
Fine needle aspiration of the specimens: cell block **A** and Diff-Quik stained section **B** from the endobronchial nodule showing mixed inflammatory cells with abundant mucin-like material. Similarly, PAP smear **C** and Diff-Quik stained section **D** of the paratracheal soft tissue nodule revealed mixture of histiocytes, lymphocytes and mucin-like material

A linear EBUS bronchoscope was then used to identify the 5 mm paratracheal soft tissue nodule (Fig. 5). Transbronchial needle aspiration was performed of the nodule and sent for cytopathology. Pathological analysis of the tracheal wall nodules (Fig. 6) as well as the paratracheal soft tissue nodule revealed mucinous-like material and chronic inflammation with focal fibrosis (Fig. 7). Neither of the samples revealed evidence of malignant cells. Cultures were finalized as negative for the growth of microorganisms after 6 weeks.

## Discussion and conclusion

Although papillary thyroid carcinoma (PTC) is the most common type of thyroid cancer, the involvement of the trachea by locoregional spread to the paratracheal space is rare [1]. In cases where PTC involves the trachea, clinical presentation can vary; airway involvement from residual disease may cause symptoms such as hemoptysis or airway obstruction, potentially leading to severe complications [10]. Standard treatment for disease involving the trachea includes procedures such as window tracheal resection and sleeve tracheal resection, both of which are associated with improved survival rates [11–13]. However, surgical resection is often reserved for larger lesions, while radioactive iodine ablation may play a role in treating smaller, residual disease in the thyroid bed and paratracheal regions.

It is important to note that the patient had spent a year living in a region of Southeast Asia with a high respiratory pathogen burden. While the submucosal nodules containing white exudate were entertained to have been from a respiratory pathogen origin, such as tuberculosis, which causes similar caseating granulomas, the patient presented negatively on all associated infectious disease pathogen screens. Histologically, the specimen from the tracheal nodule represented fibrosis and chronic inflammation. In addition, the bronchial epithelium was devoid of inflammatory cells, which further supported a noninfectious finding. Importantly, the patient did not have constitutional symptoms associated with infection, such as fevers, night sweats, unintended weight loss, cough, or dyspnea. As such, infectious etiology was deemed unlikely.

Given the anatomic location of the paratracheal nodule and the tracheal endoluminal nodules, with the same mucinous material on pathologic review from both, these findings are thought to represent previously treated sites of disease. To our knowledge, this is the first case reporting the pathologic findings of post-radioactive iodine treatment. These interesting bronchoscopic and cytopathologic findings are rare and highlight the importance of better understanding post-radioactive iodine treatment changes. This case highlights the successful treatment of radioactive iodine for follicular variant papillary thyroid carcinoma disease. Unfortunately, some patients with metastatic infiltrative disease have tumors that lose thyroid differentiation features, making the radioactive iodine refractory, thus rendering RAI treatments less effective [9, 14]. Regardless, more substantial research must be conducted to deduce the efficacy of radioactive iodine on clearing thyroid cancer tracheal metastases.

## Data Availability

Data can be shared upon reasonable request.

## Abbreviations

PTC: Papillary thyroid cancer
RAI: Radioactive
NBI: Narrow-band imaging
EBUS: Endobronchial ultrasound
